# Efficacy of sintilimab combined with neoadjuvant chemotherapy and trastuzumab in conversional treatment of locally advanced HER2-positive gastric cancer: case analysis and literature review

**DOI:** 10.1007/s00432-024-06024-6

**Published:** 2024-11-19

**Authors:** Sidikjan Ibrahim, Amina Maimaitiaili, Guangsheng Zhu, Shengwei Ye

**Affiliations:** 1 Hubei Cancer Hospital, Tongji Medical College, Huazhong University of Science and Technology, Hubei Provincial Clinical Research Center for Colorectal Cancer, Wuhan Clinical Research Center for Colorectal Cancer, Wuhan, Hubei Province China; 2 Department of Breast Surgery, The First Affiliated Hospital of Xi’an Jiaotong University, Xi’an, 710061 Shaanxi Province China

**Keywords:** Sintilimab, Neoadjuvant Chemotherapy, Trastuzumab, Gastric cancer

## Abstract

**Background:**

Regional lymph nodes that are fixed and fused into clusters or those exhibiting metastases outside the regional lymph nodes are generally classified as stage IV (M1) or unresectable. Patients with such nodes almost always need pre-operative treatment so that they can undergo surgical resection. Combining immunotherapy with trastuzumab and chemotherapy significantly improved the prognosis of HER-2 positive gastric/gastroesophageal junction (G/GEJ) cancer. However, very few reports are available on the role of immunotherapy in converting patients with unresectable cancer to resectable cancer.

**Methods:**

In this study, we report on four patients with GC who were preoperatively treated with a combination of sintilimab, trastuzumab, and chemotherapy at Hubei Cancer Hospital, China, from January 2022 to October 2023. Both preoperative and postoperative clinical and pathological characteristics of each patient were analyzed. The preoperative tumor stage was cT4N3M1.

**Results:**

Postoperative pathological results showed that two patients achieved pathological complete remission (pCR), while the pathological stage in the other two patients decreased to ypT1N0M0 and ypT2N0M0. None of them had nerve or vascular invasion. None of the patients had recurrences or metastases until the last follow-up (October 2024) after primary surgery. The present case report suggests that a combination of immunotherapy comprising trastuzumab and chemotherapy can improve the efficiency of conversion therapy for patients with HER-2 positive locally advanced G/GEJ cancer. This study also demonstrates the safety of immune checkpoint inhibitors in a conversional treatment approach.

**Conclusion:**

We showed that a pathological complete response (pCR) can be obtained even with unresectable advanced GC through treatment with sintilimab combined with neoadjuvant chemotherapy and trastuzumab.

## Introduction

Gastric cancer (GC), ranking fifth for incidence and fourth for mortality in the world, is one of the most commonly diagnosed cancers of the digestive system (Sung et al. 2021). Surgical resection is typically considered the standard of care with curative intent for GC. However, not all patients are eligible for surgical treatment upon initial diagnosis. In China, approximately 80% of patients with GC are diagnosed at an advanced stage, with a median overall survival (OS) of only 10–14 months (Fan et al. 2021; Liu et al. 2023). According to the Chinese Society of Clinical Oncology (CSCO), when the regional lymph nodes are fixed and fused into clusters, or when metastatic lymph nodes are beyond the scope of surgery, the GC is then considered unresectable. Chemoradiotherapy is generally recommended for such patients (Wang et al. 2021). If the tumor significantly shrinks after the treatment, the possibility of radical resection should be evaluated.

HER-2 is the most commonly accepted therapeutic target in advanced GC or gastroesophageal junction (GEJ) cancer (Oh and Bang 2020). Since 2010, using trastuzumab, an anti-HER-2 antibody, along with chemotherapy has become the standard first-line treatment for patients with HER-2-positive G/GEJ cancer (Bang et al. 2010). Trastuzumab induced anti-tumor effects through two ways: intracellular anti-tumor effects by inhibiting downstream effectors of HER-2 signaling, and extracellular anti-tumor effects by recruiting immunity (Oh and Bang 2020; Augustin et al. 2022). Antibody-dependent cell-mediated extracellular immunity is superior to intracellular signaling in anti-HER-2 therapy (Augustin et al. 2022). A single-arm, open-label study conducted across 11 institutions in the United states and Canada and 15 centers in Southeast Asia demonstrated the synergistic anti-tumor activity achieved through a combination of anti-HER-2 agents with anti-PD-1 checkpoint blockade (pembrolizumab) in HER-2 positive patients (Catenacci, et al. 2020). These results suggest that combining immunotherapy with anti-HER-2 therapy in GC affords some synergistic benefits.

In recent years, immune checkpoint inhibitors (ICIs) have been attracting increasing interest for cancer treatment. Considerable therapeutic effects have been achieved using ICIs in both basic and clinical investigations (Kang et al. 2017). A randomized, phase-3 trial (CheckMate 649) showed that a combination of ICI treatment with chemotherapy can reduce the risk of death by 20–35% than that achieved by using chemotherapy alone. In addition, it afforded better OS and progression-free survival (PFS) in patients with previously untreated, unresectable, non-HER-2-positive G/GEJ cancer, as well as in those with esophageal adenocarcinoma with a PD-L1 positive score (CPS) of > 5 (Janjigian et al. 2021a). Besides, two clinical trials demonstrated that a combination treatment strategy involving anti-PD1 antibody along with trastuzumab and chemotherapy provides enhanced clinical efficacy and safety for patients with metastatic HER-2-positive GC (Janjigian et al. 2020; Janjigian et al. 2021b; Lee et al. 2022). However, the effect of conversional therapy involving the use of anti-PD1 antibodies along with trastuzumab and chemotherapy in HER-2-positive GC has not yet been investigated. In this study, we present four cases of HER-2-positive GC treated through a conversional therapy involving the use of immune checkpoint inhibition along with trastuzumab and chemotherapy.

## Case presentation

Four patients were included in this study. None of them had any history of antitumor treatment. The eldest patient was 69 years old and the youngest was 54 years old. The tumor was located in the GEJ area, stomach body, and gastric antrum. All patients were diagnosed with multiple retroperitoneal lymph node enlargements. Three patients received three cycles of SOX (oxaliplatin + tegafur) combined with trastuzumab and sintilimab, while the fourth patient underwent five cycles of FLOT (docetaxel + oxaliplatin + folinate + fluorouracil) along with trastuzumab and sintilimab before surgery. A CT scan was conducted on all patients both before and after the neoadjuvant therapy. Tumor tissue was removed, washed and dried with distilled water, and the tumor tissue sections were placed on the frozen section machine table. The temperature was adjusted to −20˚C and the tissues were frozen for ~ 3 min. The diseased tissue was cut into 3‑ to 5‑μm slices, and the frozen slices were treated using a hematoxylin‑eosin staining method. After staining, the slices were placed under an optical microscope for observation at × 400 magnification at the Department of Pathology. IHC staining was conducted on a Ventana automatic immunohistochemical staining instrument (Agilent Technologies, Basel, Switzerland) using the EnVision method. The primary antibody PD-L1 (clone number SP142) was applied at a working concentration of 1:100, and the secondary antibody was used in the Optiview amplification kit (ROCHE, Basel, Switzerland). HER2 (DAKO, Basel, Switzerland, clone number EP3) was verified on the Dako automatic immunohistochemical staining instrument, and the IHC staining conditions were consistent with those of previous HER2 IHC tests. The IHC results were evaluated by two trained pathologists using a double-blind reading method, at the Department of Pathology.

### Case 1

**Fig. 1:**
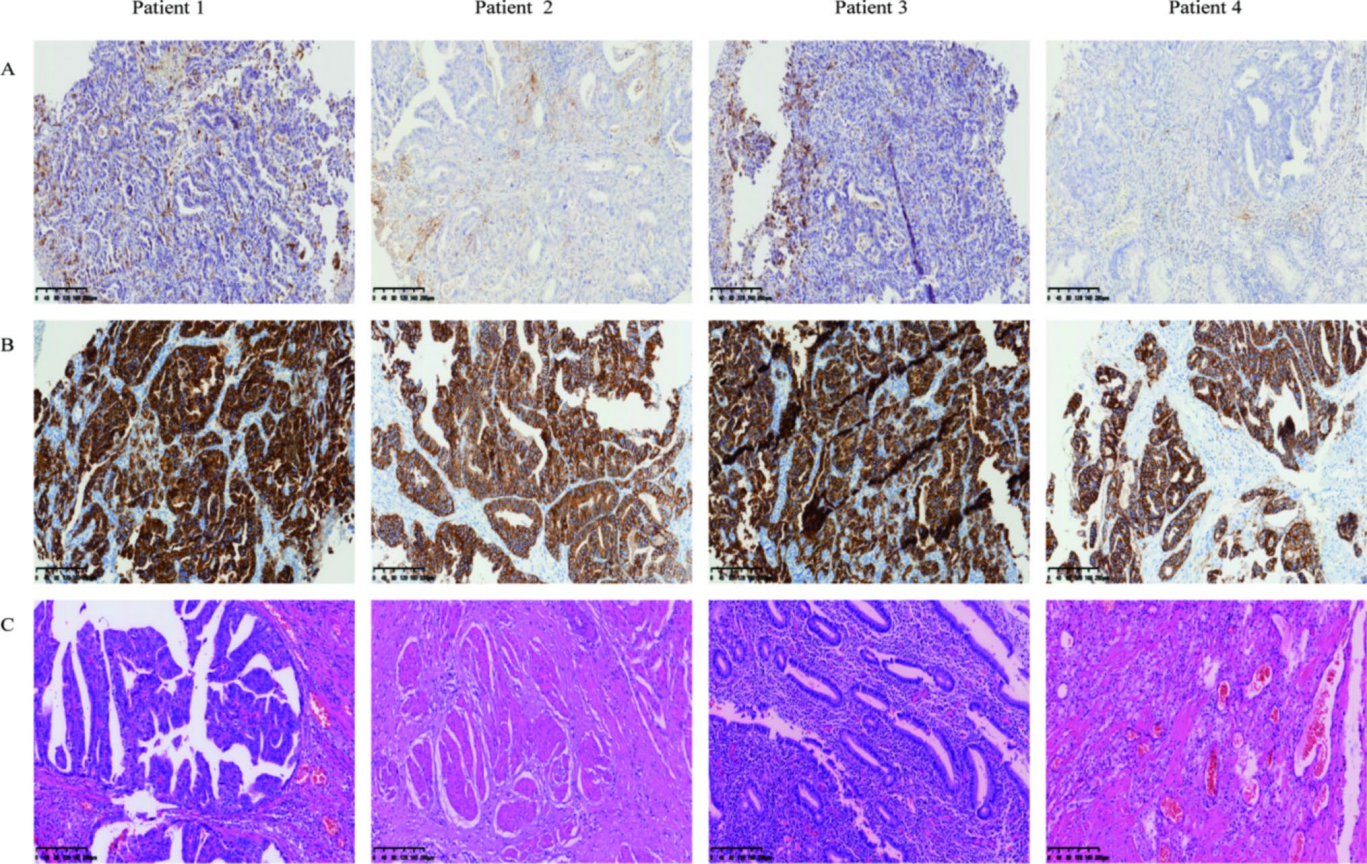
**H&E and IHC staining of patients tumor tissue.** Positive immunohistochemical staining for PD-L1 (**a**) and HER-2 (**b**) were tested in esophagocardial biopsy before neoadjuvant therapy. H&E staining were given after surgical resection for each patient after neoadjuvant therapy (**c**). Results are shown at × 400 magnification. IHC, immunohistochemical; PD-L1, programmed death ligand 1; HER2, human epidermal growth factor receptor 2

**Fig. 2:**
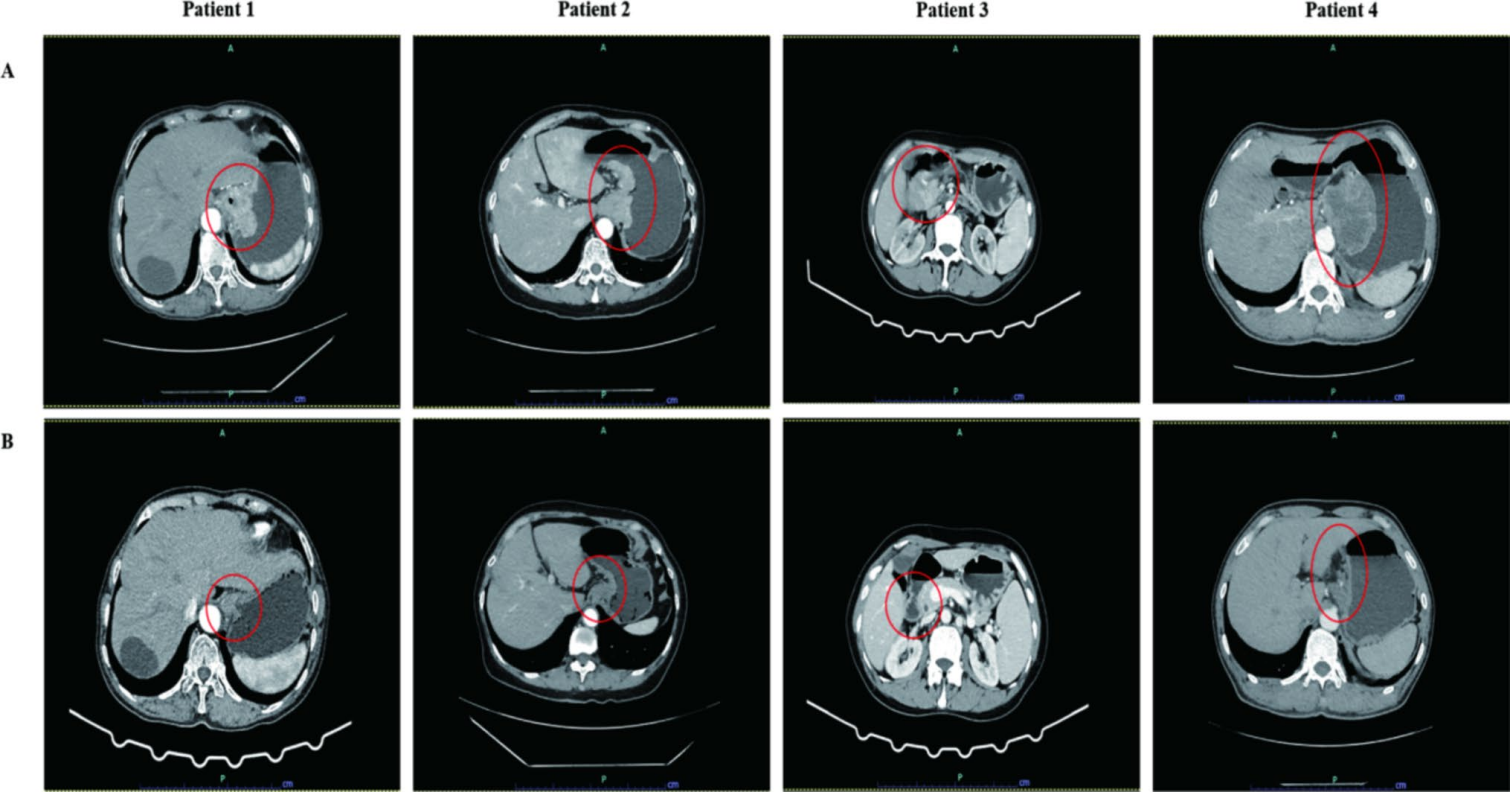
Abdominal CT scans demonstrating the disease progression following treatment**.** (**a**) CT scans at the diagnosis (before neoadjuvant therapy). (**b**) reexamination of CT scans before surgery (after neoadjuvant therapy). The red circle points out the location of the tumor. CT: Computed tomography

The specific clinicopathological information of the eldest patient is as follows: 69 years’ old, female. An esophagocardial biopsy was conducted for the patient, which revealed the following pathology results: adenocarcinoma, Her-2(3 +), PD-L1 positivity (cps = 20) **(**Fig. 1**)**. CT scans showed that the thickest part of the stomach wall was 2.5 cm. In addition, they demonstrated irregular gastric wall serosa and involvement of the perigastric fat space. Furthermore, the portal area of the liver, the hepatogastric space, the pancreatogastric space, and the retroperitoneal lymph nodes were enlarged and heterogeneously enhanced. The largest lymph node was 3.1 cm in size **(**Fig. 2**)**. After three cycles of SOX, the CA-199 level reduced from 57.88 u/ml to 30.38 u/ml **(**Table 1**)**. An analysis of the postoperative pathological results **(**Fig. 1**)** identified polypoid adenocarcinoma as the histological type. Invasion of the musculi propria by the tumor was also observed. The treatment afforded clear tumor regression, although residual cancer cells were present, mainly as single cells or in small nests. No metastatic cancer was found in the 17 lymph nodes examined. Furthermore, ypT2N0 was identified as the pathological stage **(**Table 2**)**.

**Table 1:** Comparison of tumor markers pre- and post-neoadjuvant therapy

		Pre-Neoadjuvant	Post-Neoadjuvant
Patient 1	CEA ng/ml	4.95	4.22
	CA-199 u/ml	57.88	30.38
Patient 2	CEA ng/ml	3.36	3.17
	CA-199 u/ml	9.53	8.7
Patient 3	CEA ng/ml	46	3.17
	CA-199 u/ml	1076	8.7
Patient 4	CEA ng/ml	345	5.22
	CA-199 u/ml	6.46	4.38

CEA and CA-199 were measured in each patient at the time of initial treatment (pre-neoadjuvant) and two or three days before surgery (post-neoadjuvant). CEA, carcinoembryonic antigen; CA-199, carbohydrate antigen-199

**Table 2:** Comparison of tumor stage before neoadjuvant therapy and after surgery

	Clinical stage (pre-treatment)	Pathological stage (after surgery)
Patient 1	cT4N2M1	ypT2N0M0
Patient 2	cT4N3M1	ypT0N0M0
Patient 3	cT4N2M1	ypT1N0M0
Patient 4	cT4N3M1	ypT0N0M0

cTNM classification was evaluated based on the Chinese Gastric Cancer Treatment Guidelines before any treatment (pre-treatment). ypTNM classification was evaluated according the Chinese Gastric Cancer Treatment Guidelines after radical resection (after surgery). cTNM, clinical tumor stage before neoadjuvant therapy; ypTNM, pathological tumor stage after neoadjuvant therapy

### Case 2

In the 66-year-old female patient, the biopsy of the fundus and body of stomach revealed poorly differentiated adenocarcinoma, with Her-2 (3 +) and PD-L1 positivity (cps = 13) **(**Fig. 1**)**. The CT results indicated that the thickest part of the stomach wall was 2.2 cm. The perigastric fat space demonstrated invasion by tumor cells, and multiple lymph nodes were observed along the lesser curvature of the stomach and in the retroperitoneum. The size of the largest lymph node was 1.4 cm (Fig. 1). After three cycles of SOX, the serum tumor markers reached normal levels, which did not show any significant changes during the entire treatment period **(**Table 1**)**. Postoperative pathological results **(**Fig. 1**)** revealed the tumor size to be 2 × 2 × 1.5 cm. The tumor bed was taken from a wide range of samples. A microscopic observation showed that inflammatory cells had infiltrated most of the gastric mucosa. In addition, hyperplasia of the surrounding mucosal glands was noted. A large number of foam-like histiocytes, along with inflammatory cells and multinuclear giant cells, were found in the muscularis propria. No cancer cells were found. No metastasis was found in the 28 lymph nodes. The pathological stage was identified as ypT0N0 (Table 2).

### Case 3

An antrum biopsy was conducted on the 54-year-old female patient with GC. The pretreatment pathology results indicated poorly differentiated adenocarcinoma, with Her-2 (3 +) and PD-L1 positivity (cps = 20) **(**Fig. 1**)**. After three cycles of SOX, the CA-199 level decreased from 1076 u/ml to 8.7 u/ml, while the CEA level reduced from 46 ng/ml to 3.17 ng/ml **(**Table 1**)**. The CT results showed a thickening of the antrum gastric wall and an invasion of the perigastric fat space by tumor cells. Multiple nodules and masses were observed in hepatogastric space. The space between the duodenum and colon was found to be partially fused and homogenously enhanced. The largest nodule (approximately 3.7 × 2.9 cm) was located on the lesser curvature of the stomach **(**Fig. 2**)**. Postoperative pathological results revealed the histological type to be poorly differentiated adenocarcinoma **(**Fig. 1**).** The mucous lamina propria was invaded by tumor cells. The treatment led to a significant reduction in the number of residual tumors, although residual cancer cells were still present, mostly as multiple single cells or rarely as small nests. No metastasis was found in 33 lymph nodes. The pathological stage was identified as ypT1N0 **(**Table 2**)**.

### Case 4

Cardia biopsy was conducted on the 57-year-old male patient. The pretreatment pathology results indicated adenocarcinoma, with Her-2 (3 +) and PD-L1 positivity (cps = 8) **(**Fig. 1**)**. After five cycles of FLOT, the CEA level fell down to 5.22 ng/ml from 345 ng/ml (Table 1). The CT results showed that the thickest part of the stomach wall was 6.9 cm and the largest lymph node measured 3.1 cm **(**Fig. 2**)**. Postoperative pathological results revealed necrosis of the entire area with infiltration of foam cells **(**Fig. 1**).** Histiocytes and inflammatory cells were observed. Cholesterol crystals were also seen. No clear cancer residue was detected, consistent with the complete response after treatment. No metastasis was found in 26 lymph nodes. The pathological stage was identified as ypT0N0 **(**Table 2**)**.

We observed a significant reduction in the tumor stage in two patients, while the other two patients achieved pCR. The patients who did not achieve pCR after surgery were provided with two or three cycles of the original treatment. None of them showed any symptoms of tumor recurrence until the last follow-up (October 2024).

## Discussion

According to the Chinese and Japanese Gastric Cancer Treatment Guidelines 2021, when regional lymph nodes are fixed and fused into clusters or when metastases are present outside the regional lymph nodes, the cancer is classified as stage IV (M1) or unresectable (Wang et al. 2021; Japanese Gastric Cancer Treatment Guidelines 2021). Trastuzumab combination chemotherapy is recommended as the first-line treatment of HER-2-positive unresectable advanced GC. However, combination therapy with ICIs, trastuzumab, and neoadjuvant chemotherapy is not recommended because of the lack of evidence regarding its survival benefits, as per the Japanese Gastric Cancer Treatment Guidelines 2021 (Japanese Gastric Cancer Treatment Guidelines 2021). In the present study, CT scans revealed that all patients initially presented with large huge tumors and retroperitoneal lymph node metastasis, indicating the involvement of the out-regional lymph nodes at diagnosis. None of the patients achieved R0 resection at the time of the CT scan. After 3–5 cycles of neoadjuvant therapy, the size of the tumor and lymph nodes significantly reduced. A post-therapy evaluation revealed that all patients had achieved R0 resection. The European Society for Medical Oncology Treatment Guidelines for GC also recommended preoperative chemotherapy for patients with stage IB or more advanced resectable GC (Lordick,, et al. 2022). Salah et al. studied two different groups, FLOT and ECF/ECX, undergoing chemotherapy in German hospitals from Aug 8, 2010, to Feb 10, 2015. The FLOT group exhibited a significant increase in the OS compared to that shown by the ECF/ECX group in cases of locally advanced GC (Al-Batran et al. 2019). In our study, three patients received three cycles of a combination therapy including SOX, sintilimab, and trastuzumab. The last remaining patient had a highly aggressive and large tumor, and hence was treated with five cycles of the FLOT combination therapy with sintilimab and trastuzumab. At the end of the neoadjuvant therapy, the tumor size dramatically decreased in all patients, particularly in the patient who underwent five cycles of FLOT and achieved PCR. Hence, two or three cycles of neoadjuvant therapy can be safely administered to some cases with advanced GC. Besides, different chemotherapy regimens comprising ICIs and trastuzumab afford different results. In this study, we successfully used a combination therapy involving ICIs, neoadjuvant chemotherapy, and trastuzumab in unresectable G/GEJ cancer and achieved R0 resection. In addition, two of our patients even achieved pCR.

HER-2 is overexpressed in approximately 20% of patients with GC. Its overexpression indicates aggressive cancer (Oh and Bang 2020). The success of TOGA trial showed that trastuzumab therapy can be employed to improve the clinical prognosis of patients with HER-2-positive GC (Bang et al. 2010). Subsequently, several clinical trials [e.g., HERBIS-1, WJOG7212G, CGOG1001, and KSCC (Gong et al. 2016; Yuki et al. 2020; Miura et al. 2018; Kurokawa et al. 2014)] explored the benefits of combination therapy involving trastuzumab and different chemotherapies. The results indicated that patients with GC who underwent such combination therapies exhibited a better survival rate than those who received only chemotherapy. Despite this, only a few studies are available on the use of combination therapies comprising trastuzumab and ICIs for converting unresectable GC to resectable. Our study is an attempt to fill in this gap. It shows that the combination therapy involving trastuzumab and ICIs is both highly safe and effective in unresectable advanced GC.

Trastuzumab exerts its anti-tumor effects through two pathways: intracellular and extracellular (Augustin et al. 2022). The intracellular mechanism involves inhibiting downstream signal pathways, such as pSTAT3, pAKT, and pERK, or reducing the cell-surface HER-2 phosphorylation levels through accelerated endocytic degradation (Augustin et al. 2022; Zhu et al. 2021). In the extracellular pathway, trastuzumab acts via immune antibody-dependent cell-mediated cytotoxicity (ADCC). For tumor cells treated with trastuzumab, monocytes and NK cells are the major effectors of ADCC (Augustin et al. 2022). Besides, immune activity of trastuzumab exceeds its impact upon cell signaling by changing anti-tumor immune cells activity (Augustin et al. 2022; Clynes et al. 2000; Jaime-Ramirez et al. 2011). In addition, the combination therapy involving trastuzumab and ICIs enhances HER-2-specific T-cell responses, promotes immune-cell trafficking, and induces the expansion of peripheral memory T cells (Park et al. 2010; Taylor et al. 2007; Stagg et al. 2011; Mortenson et al. 2013; Müller, et al. 2015). These results suggested that trastuzumab may exhibit a synergistic effect with ICIs. Recently, Kohei et al. showed that trastuzumab can regulate the expression of PD-L1 when HER-2-amplified GC cell lines are co-cultured with peripheral blood mononuclear cells (PBMCs) and NK cells. Immunohistochemistry analysis also suggested that trastuzumab treatment in clinical samples leads to PD-L1 upregulation (Yamashita et al. 2021). In our study, IHC confirmed that all the patients were HER-2 positive. Hence, they were administered a combination therapy comprising trastuzumab and sintilimab along with various chemotherapy regimens. All the patients were achieved R0 resection including two of them with pCR and there is no evidence of tumor recurrence when the last follow-up. The patient with the longest survival were 30 months and the patient with the shortest survival were 12 months without recurrence and metastasis.

The advent of immunotherapy has revolutionized cancer treatment. Several types of immunotherapy, including ICIs and adoptive cell transfer (ACT), have been employed to achieve reliable clinical responses. However, the efficacy of the immunotherapy varies. In addition, only a few subsets of patients benefit from it. The KEYNOTE-061 trial demonstrated that pembrolizumab did not significantly improve the OS compared to that achieved using paclitaxel in second-line therapy. However, pembrolizumab was associated with higher 24-month OS rates than those obtained using paclitaxel. Its use increased the OS even with PD-L1 enrichment among patients with PD-L1-positive GC/GEJ cancer (Fuchs et al. 2022). A phase-3 randomized clinical trial involving 763 patients, randomly assigned to pembrolizumab, pembrolizumab plus chemotherapy, or chemotherapy alone groups, demonstrated that neither pembrolizumab alone nor pembrolizumab plus chemotherapy afford superior OS and/or PFS benefits compared to that achieved with chemotherapy alone in patients with a CPS score of ≥ 1, untreated, advanced G/GEJ cancer (Shitara et al. 2020). However, the efficacy of ICIs when combined with other treatment regimens in the conversional treatment of GC, especially for unresectable GC, has not yet been clearly elucidated.

Clinical trials, such as KEYNOTE-012, ATTRACTION-2, and KEYNOTE-059, confirmed the efficacy of ICIs in the prognosis of advanced GC/GEJ cancer (Fuchs et al. 2018; Muro et al. 2016). A randomized multicenter clinical trial involving patients with untreated, HER-2-negative, unresectable advanced, or recurrent G/GEJ cancer, conducted across Japan, South Korea, and Taiwan, showed that in the nivolumab plus chemotherapy group, the median PFS was 10.45 months and the OS was 17.45 months, while they were 8.34 months and 17.15 months, respectively, in the placebo plus chemotherapy group (Kang et al. 2022). However, no study has yet reported the efficacy and safety of ICIs in the conversional therapy for GC. We recommend that the immune therapy should be used as the first-line therapy for conversional therapy. Moreover, patients with unresectable or metastatic GC with MSI-H and EBV positivity also experience significant clinical benefit from the ICI treatment, because it upregulates the PD-L1 expression (Marabelle et al. 2020; Chao et al. 2021; Rodriquenz, et al. 2020). However, not all patients reported a positive treatment outcome. Immunotherapy-induced tumor hyperprogressive disease was observed in 10% of patients with advanced GC who were treated with anti-PD-1 monoclonal antibodies (mAb) (Kamada et al. 2019). Therefore, the disease progression should be closely monitored during immunotherapy treatment.

One major limitation of our study was that the design was a single-center case analysis. In the future, more randomized prospective clinical trials are still needed to confirm the reliability and validity of our study results.

## Conclusion

The efficacy and safety of the ICI combination therapy have not yet been established in different cancers. In addition, there is a lack of research on the usefulness of conversional therapy in advanced, unresectable GC. This study presents the first case series on the application of combination therapy in the conversional treatment of GC. Further research and robust evidence are needed to validate these findings.

## Statement of ethics

The study was performed in accordance with the Declaration of Helsinki and was approved by the Ethical Review Committee of the Hubei Cancer Hospital Ethics Committee (approval no. LLHBCH2024YN-050). Since this study was retrospective and all data analysis was conducted anonymously, there was no informed consent of patients.

## Consent for publication

Not applicable.

## Funding sources

No funding support for this study.

## Data Availability

No datasets were generated or analysed during the current study.
